# EEG Microstate Differences in Medicated vs. Medication-Naïve First-Episode Psychosis Patients

**DOI:** 10.3389/fpsyt.2020.600606

**Published:** 2020-11-24

**Authors:** Amatya J. Mackintosh, Stefan Borgwardt, Erich Studerus, Anita Riecher-Rössler, Renate de Bock, Christina Andreou

**Affiliations:** ^1^Division of Clinical Psychology and Epidemiology, Department of Psychology, University of Basel, Basel, Switzerland; ^2^University Psychiatric Clinics (UPK) Basel, University of Basel, Basel, Switzerland; ^3^Department of Psychiatry and Psychotherapy, University of Lübeck, Lübeck, Germany; ^4^Division of Personality and Developmental Psychology, Department of Psychology, University of Basel, Basel, Switzerland; ^5^Faculty of Medicine, University of Basel, Basel, Switzerland

**Keywords:** electroencephalography, resting-state, schizophrenia, antipsychotic, neuroleptic, untreated, unmedicated, pathophysiology

## Abstract

There has been considerable interest in the role of synchronous brain activity abnormalities in the pathophysiology of psychotic disorders and their relevance for treatment; one index of such activity are EEG resting-state microstates. These reflect electric field configurations of the brain that persist over 60–120 ms time periods. A set of quasi-stable microstates classes A, B, C, and D have been repeatedly identified across healthy participants. Changes in microstate parameters coverage, duration and occurrence have been found in medication-naïve as well as medicated patients with psychotic disorders compared to healthy controls. However, to date, only two studies have directly compared antipsychotic medication effects on EEG microstates either pre- vs. post-treatment or between medicated and unmedicated chronic schizophrenia patients. The aim of this study was therefore to directly compare EEG resting-state microstates between medicated and medication-naïve (untreated) first-episode (FEP) psychosis patients (mFEP vs. uFEP). We used 19-channel clinical EEG recordings to compare temporal parameters of four prototypical microstate classes (A–D) within an overall sample of 47 patients (mFEP *n* = 17; uFEP *n* = 30). The results demonstrated significant decreases of microstate class A and significant increases of microstate class B in mFEP compared to uFEP. No significant differences between groups were found for microstate classes C and D. Further studies are needed to replicate these results in longitudinal designs that assess antipsychotic medication effects on neural networks at the onset of the disorder and over time during illness progression. As treatment response and compliance in FEP patients are relatively low, such studies could contribute to better understand treatment outcomes and ultimately improve treatment strategies.

## Introduction

Delusions, hallucinations, disorganized speech, catatonic behavior, and negative symptoms form the core of psychotic disorders [DSM-5®, (1)]. The lifetime prevalence of psychotic disorders is estimated at 0.75% (2). Compared to the general population, patients' life expectancy is estimated to be shortened by 15–20 years due to increased physical morbidity (3). Patients' everyday functioning such as independent living, productive activity, and social functioning is often impaired, leading to high costs beyond their medical treatment (4). Good functional outcomes were found to be related to shorter duration of untreated psychosis (5) which calls for timely and effective treatment at an early illness stage. However, discontinuation rates within 18 months of antipsychotic treatment due to inefficiency or intolerable side effects were observed to be relatively high (64–82%) in psychotic disorders (6) with a recovery rate estimated at only 13.5% (7). This clearly calls for more research on antipsychotic medication and how EEG markers and neural networks differentiate between medicated and unmedicated patients with psychotic disorders.

Electroencephalography (EEG) is one method in neuroscience research that offers several advantages. Apart from being inexpensive, non-invasive, and easy to implement, EEG can capture the fast-changing dynamics of neuronal networks with high temporal resolution in frequency bands ranging from 1 Hz to up to 200 Hz (8, 9). This allows EEG to depict coupling patterns of neural activity that might not be captured by functional Magnetic Resonance Imaging (10). There is a large body of research that has studied neuronal network disruptions in psychotic disorders using EEG methods and extensive reviews exist on the topic (11–14).

The term “resting-state” refers to intrinsic patterns of the awake state in which participants are not performing an explicit mental or physical task (15) and are postulated to show the underlying intrinsic mechanisms of the brain which influence stimulus processing as well as behavioral phenomena (16). An accumulation of evidence has shown that EEG resting-state microstates are a suitable tool to study the temporal dynamics of resting-state brain networks: EEG microstates are spatial configurations of scalp global field power that remain stable for a short period of time (60–120 ms) and occur several times per second (17, 18). These short-lasting, non-overlapping configurations of brain electric states have been divided into four prototypical microstate classes A, B, C, and D that each have a different orientation of the scalp-electric field (19): Microstate A has a left occipital to right frontal orientation, microstate B a right occipital and left frontal, microstate C a symmetric occipital to prefrontal and microstate D a symmetric frontocentral to occipital orientation (17). These four classes explain 65–84% of EEG data variance (20) and were shown to have high test-retest reliability and cross-method consistency (21). The microstate classes are described by three statistical parameters; the duration of each class in milliseconds, mean number of occurrence per second and percentage of time covered by each class (17).

Microstates were hypothesized to be the fundamental building blocks of human information processing and were found to differ across sex groups (22), over the course of development (17, 22) and between different brain states such as sleep and wakefulness (23, 24). Studies using simultaneous EEG-fMRI methods have correlated microstate classes to different resting-state networks (25, 26). Furthermore, abnormal patterns have been described in various mental conditions (27–30), most notably in psychotic disorders: Microstate differences across all classes were found for medicated (31–35), as well as medication-naïve patients with psychotic disorders (30, 36–38) compared to healthy controls, as well as patients in the high-risk state of psychosis (31, 36, 39). Two recent meta-analyses found increased occurrence of microstate C and decreased duration of microstate D to be consistently reported across studies in medicated as well as medication-naïve patients with psychotic disorders (40, 41).

So far, it is not clear whether and how antipsychotic medication treatment plays a role in EEG resting-state microstate abnormalities in patients with psychotic disorders. Antipsychotics have been shown to modulate neural networks in fMRI studies (42, 43) and to have effects on microstate parameters by increasing the mean duration of all microstate classes in healthy individuals (44). However, so far only two studies investigated the effects of antipsychotic treatment on EEG microstates in patients with psychotic disorders. A cross-sectional study from more than two decades ago with chronic schizophrenia patients reported antipsychotic treatment to be negatively correlated with microstate duration in a dose-dependent way and average microstate duration was longer in unmedicated than medicated patients (45). Moreover, increased duration of microstate classes A and D, and decreased occurrence of microstate class C was reported in responders vs. non-responder schizophrenia patients following 2–8 week treatment with antipsychotics in a longitudinal design (37). However, the latter findings were based on a small sample size (*n* = 14) and have not yet been replicated.

The present study therefore aimed to investigate the effects of antipsychotic treatment on EEG resting-state microstate parameters by comparing medicated first-episode psychosis patients (mFEP) to a control group of patients who were medication-naïve (untreated first-episode psychosis; uFEP). Our comparisons were set out to investigate differences in parameters of microstate classes A-D that might be attributed to the medication status of the two patient groups beyond the effect of the disorder. As the results of previous studies have been inconsistent so far, we based our study hypotheses on a recent study by our group (36) in which we suggested that microstate A and B may be state markers for psychotic disorders. We therefore hypothesized antipsychotics to associate with microstate A and B and these microstates to differentiate the two patient groups.

## Methods

The data used in this paper was collected in the FePsy project (***F****rüh****e****rkennung von* ***Psy****chosen*; Early Detection of Psychoses) of the University of Basel Psychiatric Clinics (UPK) during the time period from 2000–2013. The aim of the FePsy project was to improve early detection and intervention of psychosis. The study was approved by the local ethics committee and in accordance with the Declaration of Helsinki. Riecher-Rössler et al. (46, 47) provide a comprehensive overview of the FePsy study design.

### Participants

All first-episode psychosis (FEP) patients included in the present paper were help-seeking consecutive referrals to the FePsy clinic at the psychiatric outpatient department of the University of Basel Psychiatric Clinics (UPK). Upon inclusion in the FePsy study, written informed consent was given by all participating patients. The Basel Screening Instrument for Psychosis [BSIP; Riecher-Rössler et al. (47, 48)] was used to determine the FEP status, diagnostics were made according to ICD-10 (49), the Brief Psychiatric Rating Scale [BPRS; (50, 51)] was applied to assess patients' symptom severity, and the German version of the multiple choice vocabulary test [Mehrfach-Wortschatz-Test; (52)] was used to assess verbal IQ. The status of medication-naïve was defined by the absence of any lifetime antipsychotic treatment and illness duration for both groups was calculated based on the patient's reports in hindsight of the very first occurrence of psychotic symptoms with sufficient severity. As Table 1 displays, intake of other medication did however occur in the uFEP group.

**Table 1:** Sample demographics.

	**mFEP**	**uFEP**			
	***n =* 17**	***n =* 30**	**t/χ2**	***p***	***d***
Sex (M:F)	13:4	19:11	0.862	0.353	–
Age at diagnosis (years) (mean [SD])	27.68 (5.1)	28.63 (7.5)	−0.517^*^	0.608	0.148
*BPRS (mean [SD])*	47.66 (7.15)	53.68 (10.84)	−1.70	0.10	0.66
Total score					
Depression/anxiety	9.45 (3.24)	11.70 (4.39)	−1.54	0.13	0.58
Psychosis/thought disturbance	10.64 (2.46)	12.13 (3.37)	−1.33	0.19	0.50
Negative symptoms	5.41 (2.20)	5.72 (2.76)	−0.34	0.74	0.12
Activation	6.73 (3.16)	7.28 (3.50)	−0.45	0.65	0.16
Duration of illness (months) (mean [SD])	24.83 (22.61)	23.77 (35.36)	0.11	0.91	0.04
*Comorbidities (ICD-10)*			–	0.81^**^	–
F10-F19^1^	0	1			
F30-F39^1^	5	7			
F40-F49^1^	1	0			
F60-F69^1^	0	1			
CPZ equivalent dose (mean [SD])	210.29 (262.71)	n/a	–	–	–
Further medication			–	1^**^	–
Antidepressants	2	4			
Anxiolytics	4	7			
Mood stabilizers	0	0			
Other	1	2			
Current drug use			–	0.44^**^	–
Yes	11	20			
No	5	4			
Current alcohol use			–	1^**^	–
Yes	8	12			
No	8	12			
Cannabis use			–	0.52^**^	–
1)Earlier					
Yes	9	18			
No	7	5			
2)Currently					
Yes	6	9			
No	11	18			
Verbal IQ^*^ (mean [SD])	103 (16.04)	107.28 (14.34)	−0.84	0.41	0.28
School education (years) (mean [SD])	10.71 (3.25)	11.20 (3.22)	−0.50	0.62	0.15
Education level			–	0.84^**^	–
Education ongoing	2	1			
Primary school	1	1			
Secondary school	9	11			
Upper/specialized secondary school	1	2			
High school without completion	0	2			
High school	3	6			
Current employment			–	0.71^**^	–
Yes	3	6			
No	13	17			
EEG total analysis time (seconds) (mean [SD])	300.30 (74.83)	299.20 (44.72)	0.055	0.957	0.018
EEG explained variance (%) (mean [SD])	77.43 (3.36)	77.36 (3.61)	0.073	0.942	0.020

*mFEP, medicated first-episode psychosis patients; uFEP, untreated, medication-naïve first-episode psychosis patients; BPRS, Brief Psychiatric Rating Scale; CPZ, Chlorpromazine; SD, standard deviation; d, Cohen's d effect size*;

* *assessed with the German version of the multiple choice vocabulary test [Mehrfach-Wortschatz-Test; (52)]*;

** *Fischer's exact test applied*.

1 *F10-F19, Mental and behavioral disorders due to psychoactive substance use; F30-F39, Mood [affective] disorders; F40-F49, Neurotic, stress-related and somatoform disorders; F60-F69, Disorders of adult personality and behavior. Significance level is 0.05*.

Exclusion criteria were applied as follows; (1) age < 18 years; (2) insufficient knowledge of German; (3) IQ < 70; (4) serious medical or surgical illness; (5) previous episode of psychosis due to substance abuse, and (6) psychotic symptomatology within a clearly diagnosed affective or borderline personality disorder.

### EEG Recording and Pre-processing

A standard clinical EEG protocol of 20 min (incl. resting-state, eyes opening, photostimulation, and hyperventilation) was recorded by a trained lab assistant using 19 gold cup electrodes (Nicolet Biomedical, Inc.) of the International 10–20 system and referenced to linked ears. Participants were comfortably seated in a quiet room. The first 8 min of the entire clinical EEG recording corresponded to a resting-state eyes-closed recording which was used for the present analysis. During this, participants were asked to open their eyes for 6 s every 3 min to avoid drowsiness. When behavioral or EEG signs of drowsiness (e.g., slow rolling eye movements, alpha drop-out, increased beta, or theta activity) occurred, participants were asked to open their eyes. The sampling rate was 256 Hz and electrode impedances were always kept below 5 kΩ.

Brain Vision Analyzer (Version 2.0, Brain Products GmbH, Munich, Germany) was used for offline pre-processing. After bandpass (IIR; 0.5–70 Hz) and notch (50 Hz) filters were applied, eyes-open epochs and epochs with prominent muscle artifacts or bad EEG signals were removed manually upon visual inspection by trained staff. After that, interpolation was applied for channels with severe artifacts across the whole recording and Extended Infomax ICA was used to remove ocular muscle artifacts. The continuous EEG recording was then divided into 2s segments and segments with residual artifacts were removed semi-automatically and by means of visual inspection based on consensus between at least two independent reviewers. Re-referencing was applied with a common average reference and the data was finally bandpass filtered (FIR; 2–20 Hz).

### Microstate Analysis

The Microstate Analysis plug-in (Version 0.3; downloaded from http://www.thomaskoenig.ch/Download/EEGLAB_Microstates/) for EEGLAB (53) version 13.6.5b in Matlab (54) was used for the microstate analysis. First, the Global Field Power (GFP) was calculated for each time point of the recording. Since the signal-to-noise ratio is the highest for GFP peaks, microstate configurations remain stable around these peaks (17). Using Atomize-Agglomerate Hierarchical Clustering (AAHC), individual microstate maps for GFP peaks only were calculated for each participant based on the original momentary maps (55). Four microstate classes have been described to explain 65–84% (20) of the EEG variances. Based on this and for comparability with previous studies on psychotic disorders, the number of microstate clusters for the present study was also pre-set to four.

**Figure 1:**
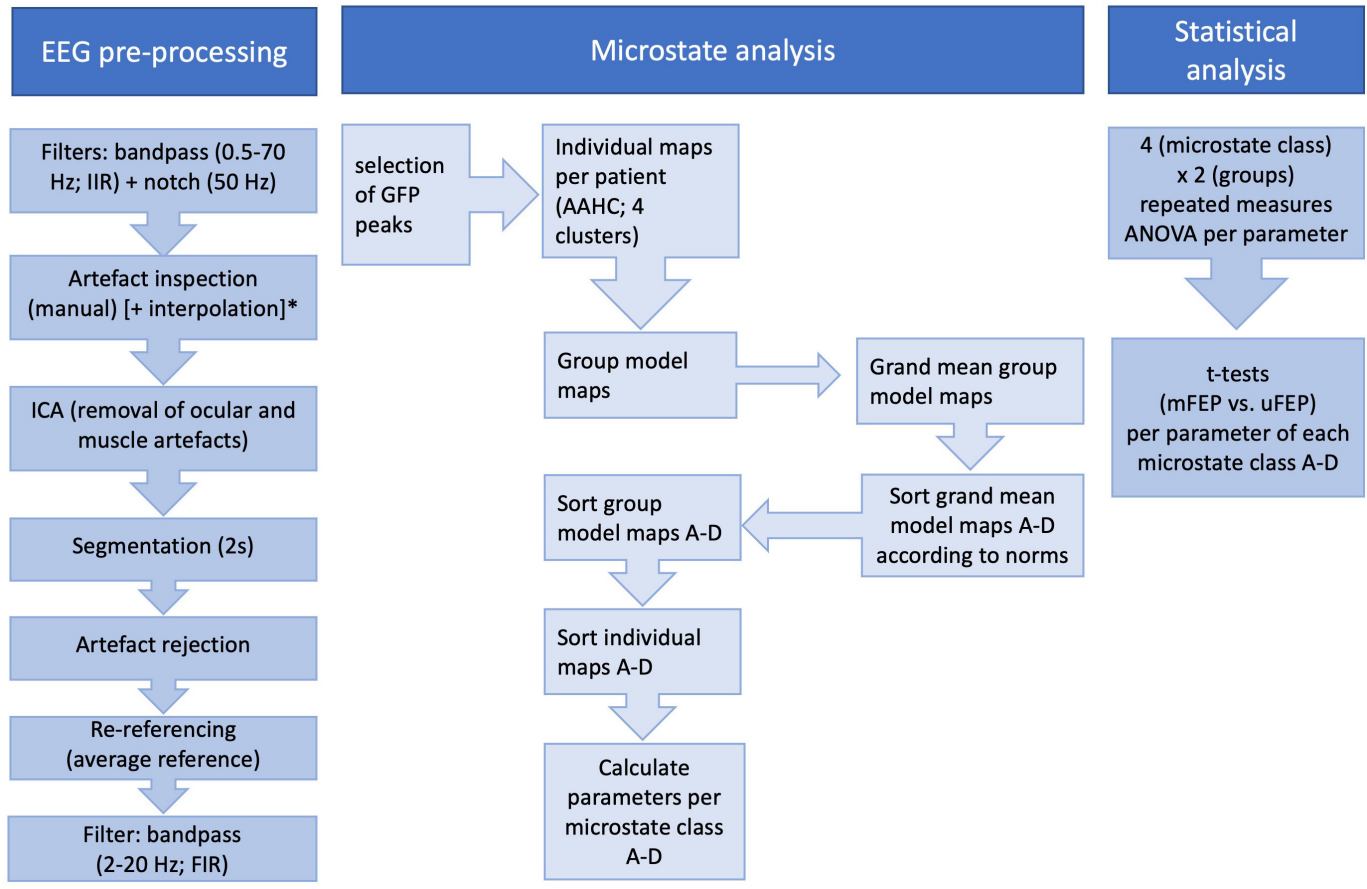
All performed steps of EEG pre-processing, microstate analysis, and statistical analysis. ICA, Independent Component Analysis; GFP, Global Field Power; AAHC, Atomize-Agglomerate Hierarchical Clustering; *Interpolation was only performed for channels with severe artifacts across the whole recording.

Group model maps were calculated separately for both patient groups using a permutation algorithm that minimized common variance across subjects (19). Based on these group models, a “grand-mean” model was calculated. The grand-mean model was then class-labeled into microstates A–D by using minimal Global Map Dissimilarity and model map norms from Koenig et al. (17). Next, the class-labeled “grand-mean” model maps were used as a template to assign the group model maps to the four class-labeled grand-mean maps. As a final step, the individual microstate maps were sorted according to the class-labeled group model maps. Three parameters were then extracted per microstate class: coverage (percentage of analysis time covered by each microstate class), duration (the average duration of a microstate class in milliseconds), and occurrence per second (total number of each microstate class per second). As the microstate toolbox ignores the first and last segment of the EEG data, only non-truncated microstate parameters are calculated. In addition, microstate transition probabilities (observed minus expected) were calculated. Figure 1 depicts all analysis steps.

### Statistical Analysis

A 4 (microstate class) × 2 (group) repeated measures ANOVA was applied to assess the interactional effect for each microstate parameter. Independent *t-*tests between the two groups (mFEP vs. uFEP) were conducted in order to determine group differences per parameter for each microstate class and demographic variables.

All analyses were carried out using SPSS 25 and R (56). Statistical tests in the present study are two-sided tests and the statistical level was set at α = 0.05. When equal variances could not be assumed, the Greenhouse-Geisser correction for ANOVAs and the Welch-Satterthwaite method for the *t-*tests was applied. Microstate results were corrected for multiple comparisons within each parameter (57).

## Results

### Group Characteristics

From a total of 59 FEP patients with available EEG data, 12 patients were excluded ex post facto due to unclear medication status. Thus, a total of 47 participants were included in the present analysis, consisting of 30 untreated, medication-naïve patients with first-episode psychosis (uFEP) and 17 medicated patients with first-episode psychosis (mFEP). There were no statistically significant differences between the two groups in age at diagnosis, sex distribution, illness duration (months), and symptom severity score as assessed with the Brief Psychiatric Rating Scale (51). Table 1 displays the demographics of the two study groups and Table 2 gives an overview of the ICD-10 diagnosis types per group which did not significantly differ between the two groups either. Approximately, a mean of 5 min resting-state recording per subject were used for further analysis (mFEP mean 300.3 s, and uFEP mean 299.2 s, respectively) which equals ~150 epochs of 2 s length per subject of each patient group. If channels were interpolated, these did not exceed a maximum of 4 channels per participant (mean 0.61, SD 1.00; range 1–4 channels).

**Table 2:** Overview of diagnosis types per group.

**Type of psychotic disorder**	**ICD-10 Code**	**mFEP**	**uFEP**	***p***
		***n =* 17**	***n =* 30**	**0.154**
Paranoid schizophrenia	F20.0	10 (58%)	14 (47%)	
Hebephrenic schizophrenia	F20.1	0	2 (7%)	
Undifferentiated schizophrenia	F20.3	1 (6%)	0	
Other schizophrenia	F20.8	1 (6%)	0	
Schizophrenia unspecified	F20.9	1 (6%)	4 (13%)	
Persistent delusional disorders	F22.0	2 (12%)	1 (3%)	
Acute and transient psychotic disorders	F23.x	1 (6%)	7 (23%)	
Schizoaffective disorder, depressive type	F25.1	0	2 (7%)	
Unspecified non-organic psychosis	F29	1 (6%)	0	

*mFEP, medicated first-episode psychosis patients; uFEP, untreated, medication-naïve first-episode psychosis patients. Significance level is 0.05*.

### Microstate Parameters: Overall Results

**Figure 2:**
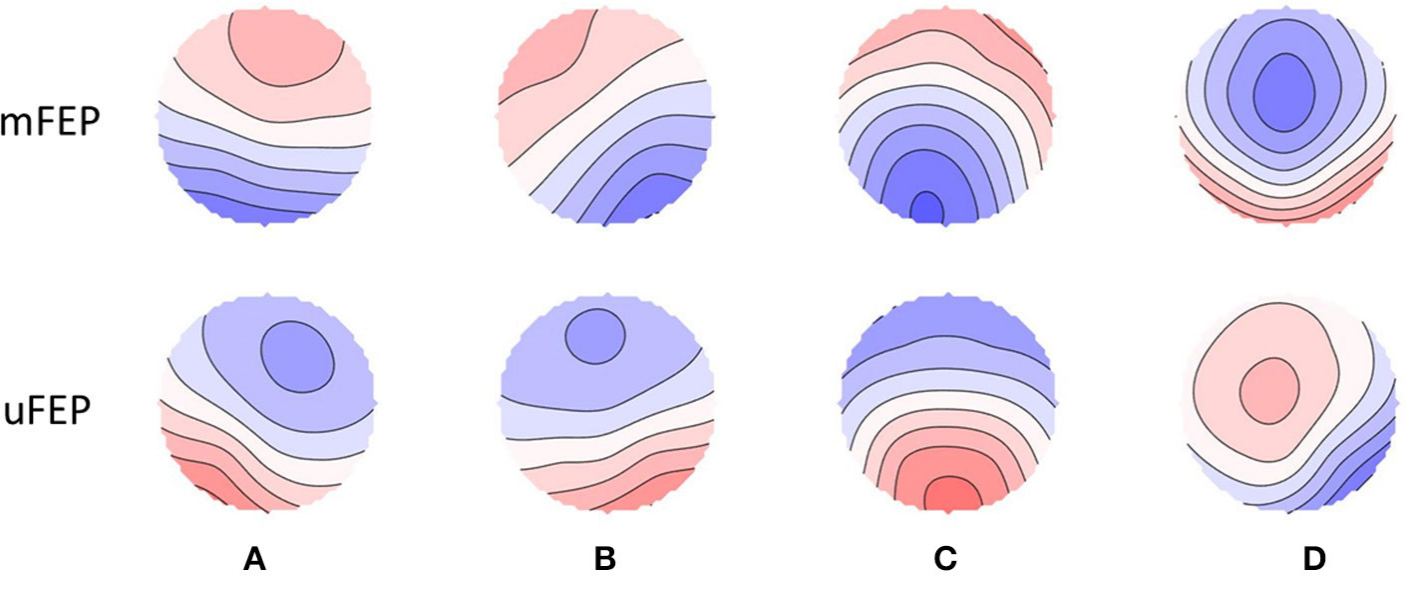
Spatial configuration of the four microstate classes. Each row displays the four microstate classes **(A–D)** for both groups. Polarity is ignored. mFEP, medicated first-episode psychosis patients; uFEP, untreated, medication-naïve first-episode psychosis patients.

Class-labeled group model maps were calculated separately for each participant group and are shown in Figure 2. The average global explained variance across both groups was 77.4% and the EEG total analysis time (seconds) did not significantly differ between groups (see Table 1).

### Microstate Parameters: Between-Group Differences

**Figure 3:**
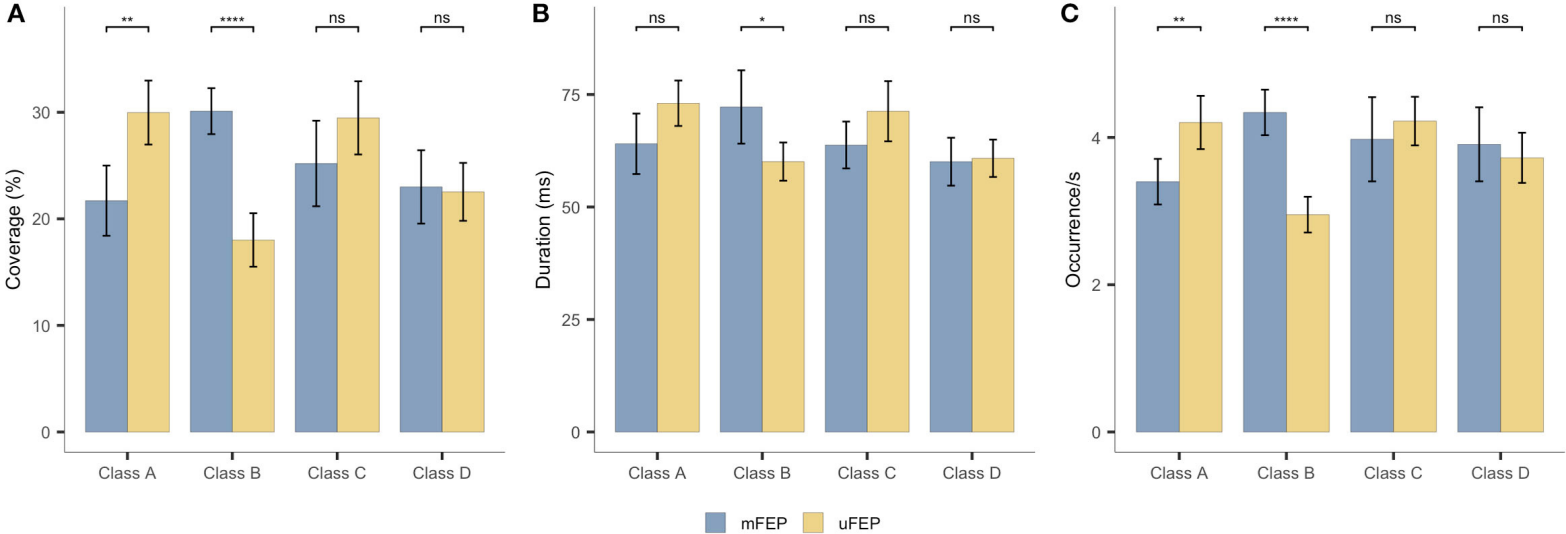
Microstates statistics. Group averages of the temporal parameters: **(A)** coverage, **(B)** duration and **(C)** occurrence. *p < 0.05, **p < 0.01, ****p < 0.0001.

The microstate class x group interactions were significant for all microstate parameters: coverage [F_(3,135)_ = 11.603, *p* < 0.001, η_p_^2^ = 0.205]; duration [F_(2.414, 108.616)_ = 7.698, *p* < 0.001, η_p_^2^ = 0.146]; and occurrence [F_(3,135)_ = 14.417, *p* < 0.001, η_p_^2^ = 0.243]. Follow-up *t-*tests indicated significant decreases of mFEP compared to uFEP for microstate A coverage [t_(39.8)_ = −3.87, *p* = 0.001, *d* = −1.14], and occurrence [t_(44.5)_ = −3.51, *p* = 0.003, *d* = −1.00]. No significant group differences were found for microstate A duration [t_(34.3)_ = −2.24, *p* = 0.094, *d* = −0.68]. Significant increases in the mFEP compared to uFEP group were found for microstate B coverage [t_(44.5)_ = 7.58, *p* < 0.001, *d* = −2.16], duration [t_(25.5)_ = 2.78, *p* = 0.040, *d* = 0.88], and occurrence [t_(35.6)_ = 7.39, *p* < 0.001, *d* = 2.22]. No significant results were found for microstate C coverage [t_(38.1)_ = −1.69, *p* = 0.198, *d* = −0.50], duration [t_(45.0)_ = −1.83, *p* = 0.146, *d* = −0.52], and occurrence [t_(27.5)_ = −0.79, *p* = 0.876, *d* = −0.25], as well as microstate D coverage [t_(35.8)_ = 0.22, *p* = 0.827, *d* = 0.07], duration [t_(35.4)_ = −0.23 *p* = 0.817, *d* = −0.07], and occurrence [t_(31.5)_ = 0.63, *p* = 0.876, *d* = 0.19]. Figure 3 and Supplementary Table 1 display means for all microstate parameters. The transition probabilities from class A to B [t_(32.0)_ = 3.97, *p* = 0.004, *d* = 1.21] and class C to B [t_(44.9)_ = 5.97, *p* < 0.001, *d* = 1.68] were increased in mFEP compared to uFEP. The transition probabilities from class A to C [t_(34.2)_ = −3.40, *p* = 0.016, *d* = −1.03] and class C to A [t_(43.7)_ = −4.69, *p* < 0.001, *d* = −1.35] were decreased in mFEP compared to uFEP. Detailed results are displayed in Supplementary Table 2.

## Discussion

We compared EEG microstate dynamics in medicated and medication-naïve first-episode psychosis patients (mFEP and uFEP, respectively). The microstate parameters coverage (%), duration (ms) and occurrence/s of four microstate classes (A–D) were compared between the two patient groups. We were able to confirm the hypothesis of an association between antipsychotics and microstate classes A and B.

We observed decreased microstate A coverage, and occurrence in mFEP compared to uFEP. This finding is underlined by a decrease of transitions from microstate C to A in mFEP compared to uFEP. Previous studies in unmedicated patients have reported an increase in microstate A compared to healthy controls (19, 30, 36, 38). Here, we show a decrease in this class in medicated patients, suggesting a beneficial association of antipsychotics with microstate A. Converging with our results, a decrease of microstate A was observed in medicated first-episode patients compared to healthy controls (34) and microstate A was positively correlated with psychopathological symptoms such as depression (28) and negative symptoms of the avolition-apathy domain (33) in patients with psychotic disorders.

Interestingly, another study observed an increase in the same microstate class in more chronic medicated patients with schizophrenia spectrum disorder with up to 10.5 years of illness duration (SD 8.7) (32). Thus, illness progression (first-episode vs. more chronic) may be an important factor to consider in future studies of medication effects. Another modulatory aspect of microstate A was demonstrated by Kikuchi et al. (37): Although there was no pre- vs. post-effect after 2–8 weeks of antipsychotic treatment—possibly due to the small sample size of *n* = 14 and relatively short follow-up intervals—they observed increased microstate A in responders vs. non-responders.

Further between-group differences were observed for microstate B in which coverage, duration, and occurrence were increased in mFEP compared to uFEP. In addition, we observed more transitions from microstates A and C to microstate B in mFEP compared to uFEP. Compared to healthy controls, previous studies in unmedicated patients showed a decrease in microstate B (30, 38, 58). Again, the present study shows an opposite effect in medicated patients which could be an indication of a positive treatment effect for this class. This is further underlined by Andreou et al. (31) in which medicated first-episode patients also showed an increase in coverage of microstate B. On the other hand, Baradits et al. (32) found a decrease in all parameters of microstate B. However, inclusion of medicated schizophrenia patients with an average illness duration of 10.5 years in the latter study could again explain the difference in findings. This is in line with the recent suggestion that microstate B might be a specific state biomarker for psychotic illness progression (36).

Despite the fact that several studies found changes in microstate C between unmedicated (30, 37, 38) and medicated patients with psychotic disorders (33, 34, 37, 59) compared to healthy controls, we did not observe any significant differences between mFEP and uFEP in the present study. The absence of a difference might be explained by the fact that previous studies have reported the same finding, i.e., increase in microstate C compared to healthy controls, regardless of whether they assessed medicated or medication-naïve patient samples. Therefore, it is conceivable that microstate C changes in patients are independent of medication status; however, larger studies are warranted to confirm our negative finding.

No significant differences in microstate D were observed between the two groups either. This is somewhat surprising, given that changes in microstate D are a central finding of studies comparing (both medicated and unmedicated) patients with psychotic disorders to healthy controls (19, 30, 32, 37, 38, 40, 59). Microstate D has further been associated with (positive) psychotic symptoms: a decrease was observed during periods of auditory hallucinations (60) and an increase in patients who responded well to antipsychotic medication (37). However, a study by Andreou et al. (31) comparing patients with FEP to a high-risk group with a similar symptom profile observed no differences in microstate D. The symptom severity scores of the patients in the present study did not significantly differ, with both groups being within the “markedly ill” range (61). This could explain why no differences in microstate D were observed. However, there is also an alternative explanation: We previously suggested that microstate D serves as a trait marker for psychotic disorders (36) in which case no effects of medication would be expected. Furthermore, a study by da Cruz et al. (40) suggested microstate D as endophenotype for psychosis in non-affected siblings of schizophrenia patients. To this end, studies with larger sample sizes are needed to further investigate medication effects on microstate D in patients with psychotic disorders.

Response status is an important issue to be considered in future studies investigating antipsychotic medication effects since it differs between individual patients (62, 63). As already mentioned, Kikuchi et al. (37) reported differences between patients that were classified as responders vs. non-responders to antipsychotic medication. However, their finding warrants replication, given that it was based on a small sample size (*n* = 7 per group). Unfortunately, it was not possible to trace response history for patients included in the present study; further studies should therefore investigate this issue. In addition, studies with longitudinal within-subject designs should explore the effects of antipsychotic medication treatment on EEG resting-state microstates, their association with individual response trajectories, as well as the role of patient baseline characteristics on medication effects. Ultimately, such studies could set the first steps into personalized medicine. This approach has been suggested for major depressive disorders and attention deficit disorders [for a review see Olbrich et al. (64)]. EEG resting-state microstates are particularly suited for this purpose, given that they have been suggested to be promising candidate biomarkers in psychotic disorders (32, 36, 40).

Further limitations of the present study have to be considered as well. Although the changes observed in medicated patients are in the expected direction, i.e., in the opposite direction of changes reported in previous studies comparing unmedicated patients to healthy controls, the inclusion of a matched healthy control sample would have been advantageous in completing the picture. Besides a healthy control group, a longitudinal design would have enabled us to confirm that the observed effects in medicated patients indeed correspond to a “normalization” of microstate parameters. A larger sample size than the one used here would have further increased statistical power of the results. Studies with high power are more likely to find true effects, e.g., correlation coefficients are estimated with a higher precision when sample sizes are increased (65). Moreover, it is due to the small sample size that we could not explore correlations between the four factors of the BPRS (with which the patients' symptom severities were measured) and the three parameters coverage, duration and occurrence of each microstate class A, B, C, and D. This could therefore be considered as a further limitation of this study.

In addition, cautiousness is warranted in the interpretation of our results, as a decrease or increase of a given parameter does not necessarily correspond to a “good” or “bad” outcome. Previous studies comparing first-episode patients (FEP), ultra-high-risk for psychosis patients and/or unaffected siblings of patients, and healthy controls have demonstrated that microstate changes do not always follow a linear pattern across different stages of psychotic disorders (31, 36, 40). Moreover, it has been suggested that some of the observed changes may reflect compensatory mechanisms rather than a deficit (31, 40). A further limitation of our study regards information which was not known for our sample and could have acted as confounding factor. This includes potentially different effects of individual antipsychotics (i.e., first vs. second generation antipsychotics) on EEG, medication duration, antipsychotic side effects, medication compliance, markers of socio-economic functioning, nicotine use, as well as the time of day of the EEG recording. Further confounding factors could have been age and sex distributions, as well as illness duration, drug consumption or other medication. However, all these variables did not significantly differ between groups.

Furthermore, two methodological points should be considered as well. First, based on previously established norms by Koenig et al. (17) the present study assessed four microstate classes. However, as suggested by Custo et al. (26) an increased number of microstates with a 7-map model might improve the explained global variance (20). Nevertheless, using four microstate classes has the important advantage of allowing direct comparisons of our results with previous studies in patients with psychotic disorders and high psychosis risk. Together with our relatively high global explained variance of 77%, we therefore deem our current method appropriate. As a second methodological limitation, it should be kept in mind that different pre-processing strategies, data selection methods and smoothing parameters (20) as well as differences in microstate analysis steps [e.g., the template used for microstate class assignment (21)] may influence microstate temporal parameters. In our study, we chose pre-processing and analysis parameters such as to ensure maximum comparability with a previous study by our group (36) but there may be differences compared to other studies. For future research in the field of EEG microstates, it would be very useful to harmonize methods in order to promote comparability.

## Conclusion

Our findings suggest an association of antipsychotic medication with microstates A and B in first-episode psychosis patients. Further studies with large sample sizes and longitudinal designs are needed that directly compare medicated and medication-naïve patients as well as healthy controls, in order to investigate antipsychotic medication effects on neural networks over time and throughout illness progression.

## Data Availability Statement

The raw data supporting the conclusions of this article will be made available by the authors, without undue reservation.

## Ethics Statement

This study involving human participants was reviewed and approved by EKNZ Basel. The patients provided their written informed consent to participate in this study.

## Author Contributions

AR-R was responsible for the conception and design of the FePsy study. CA and SB contributed to the acquisition of data. CA, RB, and AM were responsible for the conception and design of the current microstate analysis whilst. AM, RB, and ES performed the statistical analysis. AM and RB wrote the first draft of the manuscript. All authors contributed to critical revision for important intellectual content and final approval of the submitted manuscript.

## Conflict of Interest

The authors declare that the research was conducted in the absence of any commercial or financial relationships that could be construed as a potential conflict of interest.
