# Supplementary material for: EEG Microstate Differences in Medicated vs. Medication-Naïve First-Episode Psychosis Patients
Source: Front Psychiatry. 2020 Nov 24;11:600606. doi: 10.3389/fpsyt.2020.600606 (PMC7732503; doi:10.3389/fpsyt.2020.600606)
Supplement: Supplementary file 1 [file Data_Sheet_1.docx]

| Supplementary Table 1. Means for all microstate parameters. | | | | | |  | | |  |  |  |
| --- | --- | --- | --- | --- | --- | --- | --- | --- | --- | --- | --- |
|  | **mFEP** | **uFEP** | **t** | **p** | **effect size** | | | **direction** | | | |
| *Coverage (%) (mean[SD])* |  |  |  |  |  | |  | | | |  |
| A | 21.707 (6.406) | 29.974 (8.038) | -3.87 | 0.001 | -1.14 | | mFEP < uFEP | | | |  |
| B | 30.105 (4.194) | 18.018 (6.722) | 7.58 | <0.001 | 2.16 | | mFEP > uFEP | | | |  |
| C | 25.192 (7.806) | 29.474 (9.199) | -1.69 | 0.198 | -0.50 | |  | | | |  |
| D | 22.995 (6.692) | 22.533 (7.278) | 0.22 | 0.827 | 0.07 | |  | | | |  |
| *Duration (ms) (mean[SD])* |  |  |  |  |  | |  | | | |  |
| A | 64.046 (13.075) | 73.061 (13.518) | -2.24 | 0.094 | -0.68 | |  | | | |  |
| B | 72.230 (15.847) | 60.092 (11.373) | 2.78 | 0.040 | 0.88 | | mFEP > uFEP | | | |  |
| C | 63.779 (10.132) | 71.283 (17.908) | -1.83 | 0.146 | -0.52 | |  | | | |  |
| D | 60.073 (10.349) | 60.825 (11.110) | -0.23 | 0.817 | -0.07 | |  | | | |  |
| *Occurrence/s (mean[SD])* |  |  |  |  |  | |  | | | |  |
| A | 3.399 (0.602) | 4.203 (0.968) | -3.51 | 0.003 | -1.00 | | mFEP < uFEP | | | |  |
| B | 4.339 (0.601) | 2.951 (0.650) | 7.39 | <0.001 | 2.22 | | mFEP > uFEP | | | |  |
| C | 3.975 (1.112) | 4.222 (0.883) | -0.79 | 0.876 | -0.25 | |  | | | |  |
| D | 3.907 (0.978) | 3.724 (0.912) | 0.63 | 0.876 | 0.19 | |  | | | |  |
| *Note:* mFEP = medicated first-episode psychosis patients; uFEP = unmedicated first-episode psychosis patients; SD = standard deviation; ms = milliseconds, s = seconds; significance level is 0.05 and corrected for multiple comparisons. | | | | | | | | | |  |  |
|  |  |  |  |  |  |  |  |  |  |  |  |

| **Supplementary Table 2. Means for all transition probabilites (delta; observed minus expected).** | | | | | | | | |
| --- | --- | --- | --- | --- | --- | --- | --- | --- |
|  | **mFEP** | | **uFEP** | |  |  |  |  |
| Transition | mean | sd | mean | sd | t | p | effect size | direction |
| A-B | -0.004 | 0.007 | -0.012 | 0.007 | 3.967 | 0.004 | 1.212 | mFEP > uFEP |
| A-C | -0.001 | 0.007 | 0.006 | 0.008 | -3.397 | 0.016 | -1.027 | mFEP < uFEP |
| A-D | -0.003 | 0.006 | -0.005 | 0.007 | 0.898 | 1.000 | 0.265 |  |
| B-A | -0.010 | 0.007 | -0.007 | 0.007 | -1.129 | 1.000 | -0.345 |  |
| B-C | 0.000 | 0.007 | -0.001 | 0.008 | 0.420 | 1.000 | 0.125 |  |
| B-D | -0.001 | 0.007 | 0.000 | 0.008 | -0.321 | 1.000 | -0.096 |  |
| C-A | -0.004 | 0.006 | 0.006 | 0.009 | -4.694 | 0.000 | -1.347 | mFEP < uFEP |
| C-B | 0.001 | 0.004 | -0.008 | 0.007 | 5.971 | 0.000 | 1.684 | mFEP > uFEP |
| C-D | -0.007 | 0.006 | -0.009 | 0.007 | 0.815 | 1.000 | 0.239 |  |
| D-A | -0.005 | 0.005 | -0.002 | 0.007 | -1.906 | 0.443 | -0.548 |  |
| D-B | 0.001 | 0.005 | -0.004 | 0.006 | 2.770 | 0.068 | 0.820 |  |
| D-C | -0.005 | 0.006 | -0.004 | 0.007 | -0.431 | 1.000 | -0.129 |  |
| Note: mFEP = medicated first-episode psychosis patients; uFEP = unmedicated first-episode psychosis patients; sd = standard deviation; significance level is 0.05 and corrected for multiple comparisons. | | | | | | | | |
